# Exploring the impact of common assessment instrumentation on communication and collaboration in inpatient and community-based mental health settings: a focus group study

**DOI:** 10.1186/1472-6963-14-457

**Published:** 2014-10-03

**Authors:** Lynn Martin, John P Hirdes

**Affiliations:** Department of Health Sciences, Lakehead University, 955 Oliver Road, Thunder Bay, ON P7B 5E1 Canada; School of Public Health and Health Systems, University of Waterloo, 200 University Avenue West, Waterloo, ON N2L 3G1 Canada

**Keywords:** Integrated care, Communication, Collaboration, Assessment, Mental health, interRAI, RAI mental health, interRAI community mental health

## Abstract

**Background:**

Recognition that integrated services can lead to more efficient and effective care has made the principle of integration a priority for health systems worldwide for the last decade. However, actually bringing fully integrated services to life has eluded most health care organizations. Mental health has followed the rule, rather than the exception, when it comes integrating services. The lack of effective mechanisms to evaluate the needs of persons across mental health care services has been an important barrier to communication between professionals involved in care. This study sought to understand communication among inpatient and community-based mental health staff during transfers of care, before and after implementation of compatible assessment instrumentation.

**Methods:**

Two focus groups were held with staff from inpatient (n = 10) and community (n = 10) settings in an urban, specialized psychiatric hospital in Ontario (Canada) – prior to and one year after implementation of compatible instrumentation in the community program. Transcripts were coded and aggregated into themes.

**Results:**

Very different views of current communication patterns during transfers of care emerged. Inpatient mental health staff described a predictable, well-known process, whereas community-based staff emphasized unpredictability. Staff also discussed issues related to trust and the circle of care. All agreed that compatible assessments in inpatient and community mental health settings would facilitate communication through use of a common assessment language. However, no change in communication patterns was reported one year post implementation of compatible instrumentation.

**Conclusions:**

Though all participants agreed on the potential for compatible instrumentation to improve communication during transfers of care, this cannot happen overnight. A number of issues related to trust, evidence-based practice, and organizational factors act as barriers to communication. In particular, staff noted the need for the results of comprehensive mental health assessments to be transformed into meaningful, user-friendly clinical summaries to facilitate uptake of assessment information, and consequently use of a common assessment language across mental health settings.

## Background

Recognition that integrated services can lead to more efficient and effective care has made the principle of integration a priority for health systems for the last decade. However, actually bringing fully integrated or coordinated services life has eluded most health care organizations. Differentiation, or specialization, which characterizes the health care system (and is considered among its strengths), also acts as a powerful barrier to integration [1]. As a subset of the larger health care system, mental health services have followed the rule, rather than the exception, when it comes to integration across the care continuum.

Key to successful service integration are collaboration and communication among those providing care. In their review, Craven and Bland [2] found that several factors are key to successful collaboration in mental health care, including supportive structures, organization re-organization, and buy-in from staff. These authors reported that shared assessment, decision-making, and treatment planning also contribute to high levels of collaboration among staff. These findings were reinforced in a review by Lee, Crowther, Keating, and Kulkarni [3] - they reported that communication between staff was enhanced through use of shared assessments and care plans.

In 2005, the Ontario (Canada) Ministry of Health and Long-term Care (MOHLTC) mandated the use of the Resident Assessment Instrument – Mental Health (RAI-MH) in all adult inpatient psychiatric beds/facilities [4]. This represented an important first step toward a better understanding of needs, service use, quality, and outcomes of care in inpatient mental health settings. The RAI-MH is part of a larger suite of instruments developed by interRAI [5–7]; this organization released a ‘sister’ instrument to the RAI-MH designed for community mental health settings (interRAI Community Mental Health or interRAI CMH) [8–10] in 2007, but is not yet widely used in the province (though it has recently been mandated in Newfoundland and Labrador). Therefore, Ontario has no mechanism in place to consistently evaluate and track the strengths, preferences and needs of persons across the continuum of mental health services. Given that the public, private (e.g., drug costs, disability and WSIB claims) and economic (e.g., loss of productivity, involvement with the criminal justice system, accidents/damages) costs of mental health and addictions are estimated at approximately $39 billion a year in Ontario [11], there is considerable urgency for this situation to change.

The goal of this study was to understand how clinical staff in inpatient and community-based mental health settings collaborated and communicated with one another over the course of transfers of care, and to examine whether use of compatible assessment instrumentation - specifically the RAI-MH and interRAI CMH, positively affected collaboration and communication.

## Methods

### Methodology

A qualitative methodology was utilized. Specifically, focus groups were selected as the method of data collection as they are effective in exploring perspectives and views of persons with similar experiences [12]. The study received ethics approval from the Research Ethics Board at Lakehead University, as well as by the Research Ethics Board in the participating organization. The study methodology adheres to the RATS guidelines for qualitative research.

### Sampling

Participants included 20 staff from inpatient (n = 10) and community (n = 10) settings in an urban, specialized psychiatric hospital in Ontario. Staff from these two specific settings were recruited for the study given that they had frequent interactions with one another - i.e., clients in the community-based programed were referred from the inpatient setting, and most clients had multiple inpatient admissions. With the exception of one male in the community setting, all participants were women. In the inpatient setting, all participants were nurses, whereas there was more diversity in the professions of community-based staff (i.e., nurses, occupational therapists, and social workers). While the disciplines represented in the sample represent a subset of those involved in care (e.g., does not include physicians, psychiatrists, pharmacists), the sample is reflective of personnel in both the inpatient and community-based samples most often responsible for initial assessment and arranging/participating in transfers of care.

### Recruitment

Staff in both settings were recruited by the RAI-Mental Health Coordinator for the organization. This person attended a staff meeting on the inpatient unit and at the community-based program to inform them about the study and invite them to participate. Interested staff were provided with the date and time of the focus group; focus groups were held in a room on the unit for inpatient staff and in a meeting room in the community program. Prior to beginning the focus group, the study’s Information Letter was reviewed by the lead author. Once all questions about the study had been answered, staff provided written consent to participate.

### Data collection

Two focus groups were held with staff in each setting – one prior to the implementation of the interRAI CMH assessment system in the community-based program, and the other one year after its implementation. A pre-post design was used to understand the current status of communication during transfers and then to assess whether these had changed after implementation of the interRAI CMH assessment instrument. Note that the study’s Information Letter was again reviewed and consent sought at the post-implementation focus group. All discussions were audio taped and later transcribed for analysis.

In the pre-implementation focus group, the topics discussed included staff’s perceptions of:

- current collaboration and communication patterns during transfers of care (i.e., inpatient to community, or community to inpatient)
- barriers to communication and collaboration
- perceived impact of use compatible assessment instrumentation on communication and collaboration

Post-implementation, the focus was on the actual impact of compatible assessment instrumentation on communication and collaboration between settings. Ways in which communication and collaboration could further be enhanced were also discussed.

### Role of researchers

The focus groups were led by the first author (Martin), who had previously participated in the development of the interRAI CMH, as well in projects related to the RAI-MH. In this role, the author had developed a relationship with the RAI-MH Coordinator at the participating organization, who aided in recruitment of staff. The author did not have a relationship with the participants. In the community-based setting, a couple of the participants were aware of the author’s involvement in the development and pilot testing of the interRAI CMH several years earlier, and one had been involved in that pilot study.

### Analysis

Discussions were audio taped and later transcribed for analysis. The transcripts were independently coded by two researchers. Individual codes were assigned to give meaning to text, and these were agreed upon by the coders. The individual codes were aggregated into larger clusters of ideas, and supporting quotes were identified to illustrate the themes [13].

## Results

### Pre-implementation communication and collaboration during transfers of care

Very different themes emerged related to current communication patterns during transfers of care. Inpatient staff described a predictable, well-known process for communicating with staff in community settings: “We call them right away…there is that immediate notification.” In particular, inpatient staff discussed a protocol for notifying community-based staff prior to discharge: “Well, we always have the outpatient follow-up set up before we discharge patients. So we work directly with them before we discharge.” The inpatient staff also alluded to the fact that the community-based program had its own way of assessing individuals: “..so they usually come and meet with us before so that they know who they’ll be seeing and they can start their own forms”. One participant also alluded to the isolated nature of planning by community staff: “…they start working up what the treatment is going to be and how it’s going to be followed.” When asked about the transfer of information about the person, inpatient staff commented that the inpatient assessments are available to the community staff upon the person’s discharge. Staff also noted that they: “call for all of their old information and we would get that over as quickly as possible so that we know as much as possible about the person as we can.” Therefore, inpatient staff believe that a standardized protocol was in place to notify community-based staff of new clients or if their existing clients had been hospitalized; that inpatient documentation was made available to community-staff; and that they requested and reviewed records from the community program immediately upon admission, when such information existed.

However, community staff emphasized the unpredictability of communication during transfers: “sometimes it’s by accident that you find out that someone has been admitted”, and “I think it depends on the day and who you’re interacting with at the other end.” They also noted that there was also often a delay between admission of a client and notification of the community-based program staff: “…sometimes we don’t find out until weeks down the road”. Sometimes that communication didn’t happen at all: “I think there’s an expectation of communication that should happen…but unfortunately…I mean, that’s always a quality improvement that we need to look at” and “So no matter what protocols you have in place…you may not have time to observe all the protocols. That’s just the way it goes”. In terms of information sharing, community staff also had a different perspective on what was available to them, if anything: “Hopefully, you get the discharge summary and hopefully you get the list of their current meds that they were discharged on. But again, that doesn’t always happen and you have to make a special effort to obtain that”.

### Pre-implementation identification of barriers to effective communication and collaboration

Staff in both settings commented on the many issues that affected communication patterns, including a lack of understanding of the roles of professionals in the other setting (e.g., functions, expectations); difficulties surrounding the transfer of care (e.g., when does ‘our’ patient become ‘their’ patient?); system pressures (e.g., need to free up beds); and lack of resources (e.g., limited staff, high workload).

Several unique issues were also identified. Inpatient staff raised the issue of trust in the information provided by community staff: “…the information that you get…I think to kind of guarantee admission, is all the negative stuff that’s happening without it being balanced with whatever strengths are for the client.” For their part, community staff discussed exclusion from the circle of care as the main barrier to communication: “I have been told on several occasions… they can’t talk to you because you’re not in the circle of care. And then you have to, you know, call them back and say ‘Well, excuse me, but we are. We’re a part of the same organization’.”

### Pre-implementation thoughts on the potential impact of compatible assessment instruments

Staff in both settings expressed that use of compatible instruments would facilitate communication. For example, inpatient staff commented that “it would make sense to have a common language”, while community staff noted “there’s tremendous potential with this tool in terms of enhancing communication” and “…it’s something that’s structured and consistent and that talks to all of the others that might be involved with the person.” The potential benefit to patients was also expressed: “I think from the client perspective…the potential for needs to be forwarded to the next clinical team, and not having to reinvent the wheel and ask all the same questions…would help with transfer of care.”

Both groups noted that the use of compatible instrumentation could also ameliorate current communication difficulties. Inpatient staff thought that compatible instrumentation would help to build trust: “…there’d be a structured instrument like this that tells no lies, you know”, and “there is some trust between the program areas that the information is as valid…and that it can be trusted.” Community staff thought it would promote their inclusion in care: “I think that circle becomes a much more solid linkage”, and “we have the same form, so we must be on the same team.”

### Post-implementation communication and collaboration

Both the inpatient and community staff discussed the impact of compatible assessment instrumentation on collaboration and communication patterns in terms of challenges and potential solutions.

#### Challenges

While different reasons were given, staff in both settings acknowledged that use of compatible instrumentation had not yet changed communication patterns. For example: “I don’t think the RAI has been integrated into that communication”, and “even though it’s compatible…nobody’s talking about Depression Rating Scale scores or anything like that.”

Though the RAI-MH has been mandated since 2005, inpatient staff commented that they’d been “…using [it] for five years and now ‘using it’”. Ensuing discussion revolved around how their continued difficulty in integrating the vast information provided by the RAI-MH into their clinical practice likely contributed to the absence of change in communication patterns with community staff. Further, inpatient staff noted that not all staff were equally familiar with the RAI-MH, making it more difficult to incorporate its content into multidisciplinary team meetings: “We still have a ways to go in involving other multi-disciplines…but that’s coming” and “I think we’ve started involving some of the multidisciplines [sic]…some of our social workers have really been working hard at becoming involved in the RAI….physicians have been working at becoming involved.”

Community staff noted some of the challenges associated with implementation of a new assessment form or procedure (e.g., time to complete assessments and to get used to new software). Because the interRAI CMH encourages the use of a ‘lead’ assessor who takes responsibility for completing the assessment (with the help of other staff, the person, available documentation, etc.), community staff talked about the challenges associated with assessment of issues outside of their discipline. They also echoed some of the sentiments of inpatient staff by expressing that most of the previous year had been spent getting used to completing the assessment, and less on how to use the information it generated.

#### Potential solutions

Staff in both settings acknowledged that change in how they used the RAI-MH and interRAI CMH assessments was needed, and talked about ways this could be facilitated. In particular, they mentioned the need for more training on how to retrieve and interpret the information generated from the assessments: “I think there needs to be more education of the staff in integrating between [name of community program] and inpatient information, on how to utilize the two more efficiently.”

The way in which the assessment information was being provided was thought to contribute to the difficulty with its uptake: “I find that working with the tool itself is too much information and it’s too hard to find things.” Staff suggested that if the information generated by the assessment was “more useable” to the people using it, the instrument would be seen less as “a tool that people are inputting data into as we’re required to do it” and more as something that they can use in clinical decision-making. There is a need for a more meaningful and user-friendly way of summarizing the assessment information: “I think the idea of putting it in a narrative, which is more of a language of clinical relationships we have – I think it’ll be more user-friendly on the ground.”

## Discussion

The goal of this study was to understand how clinical staff in inpatient and community-based mental health settings currently communicated and collaborated with one another during transfers of care, and whether use of compatible assessment instrumentation affected these. In this study, the compatible instruments were the RAI-MH and interRAI CMH, from the interRAI suite (see http://www.interrai.org).

Overall, very different views of current communication patterns emerged, though there was substantial agreement among staff in both settings that compatible assessments would facilitate communication through use of a common language. For inpatient staff, it also meant the potential for increased trust in the information received and less of a desire to re-collect the same information for themselves. For staff in the community, it meant the possibility of being acknowledged as part of the care team by professionals in the organization and getting the information they need when they need it.

One-year post implementation of the interRAI CMH, neither group reported a change in their communication patterns. That there was not an immediate impact is neither surprising nor unexpected. It is well-known, for example, that the impact of new funds into the mental health system may not be evident until several years later [14]. Therefore, it may be some time before the impact of an integrated mental health information system on communication is realized. As this study focused on two specific instruments (i.e., RAI-MH and interRAI CMH), future work should seek opportunities to explore the extent to which other jurisdictions use alternative compatible instrumentation technologies and their impact on staff communication. Further, work that examines the impact of the RAI-MH and interRAI CMH on staff communication in other jurisdictions using both instruments should also be undertaken.

In spite of the absence of impact in using compatible instrumentation, participants did not attribute the absence of change in communication patterns to the instrumentation, but rather to their own uptake of the information it generated. Therefore, the difficulty integrating the clinical information available in the RAI MH and interRAI CMH into decision-making and practice in both settings contributed to the assessment information not being part of the communication between professionals.

In spite of this, both groups were adamant in their belief that an integrated assessment information system would improve communication and collaboration between inpatient and community staff. Both groups also expressed a strong desire for continued use of the interRAI CMH within the organization. Administration also expressed willingness to continue to support this initiative, and to expand it as well.

While the RAI-MH is completed by staff in all inpatient psychiatric facilities and units in the province, the information that it generates may not yet be used to its full capacity to support clinical decision-making. For example, the instruments both include several embedded scales (e.g., Depression Rating Scale, Cognitive Performance Scale), but these were not necessarily available to staff upon completion of the instrument. In spite of being comprised of 300+ items that inform clinical decision-making (e.g., presence of specific symptoms, cognitive functioning, etc.), the inability to quickly summarize the relevant information in the RAI-MH led some to perceive it as an administrative tool, rather than a clinical assessment. Study participants noted that if the information they needed from the RAI-MH and interRAI CMH was available in a way that was easier for them to use, it would figure more prominently in daily communication between staff as well as with professionals in other settings in instances of shared care or transfer of care. The full potential of use of a common language between all mental health professionals to promote seamless, integrated care could then be realized. Future work should be conducted to develop a summary (or other form of assessment output) and then to test its usability and ability to improve uptake of assessment information within settings, as well as the sharing of assessment information across settings.

## Conclusions

The importance of good communication and collaboration among inpatient and community-based mental health staff to providing integrated care and services is known. The introduction of compatible assessment instrumentation has been identified in the literature as a means of improving communication and collaboration, and is perceived by staff as being helpful. However, the impact of implementing compatible instruments is not immediate, and likely cannot alone improve communication and collaboration.

Both the RAI MH and the interRAI CMH include several hundred items on key life areas (e.g., psychiatric symptoms, social support, relationships, physical health, life events, employment, activities) that are relevant to the various disciplines involved in care (e.g., psychology, psychiatry, nursing, medicine, social work, vocational rehabilitation, recreation therapy). There is indeed great potential for these instruments to promote interdisciplinary mental health care. However, work is needed to organize the assessment information in a way that is useful and meaningful to the clinician who is using it. This is certainly worth doing given the recognized need for an integrated information system based on individual-level data that would permit tracking of individuals across the mental health care continuum [14], like the RAI-MH and interRAI CMH. Future work, therefore, should focus on ways to facilitate uptake of assessment information - for example, on development of evidence-based, meaningful clinical summaries for the various professionals involved in mental health care. In turn, this would promote use of a common language among mental health professionals, thus improving communication and collaboration in inpatient and community-based mental health settings.
